# Exploring barriers and facilitators for non-communicable disease care for persons living with HIV in Peru through a socioecological framework: HIV physician and coordinator perspectives

**DOI:** 10.1371/journal.pgph.0006608

**Published:** 2026-09-25

**Authors:** Rebecca Slotkin, Daniel Granda, Joselito Malca Hernandez, Diego Cabrera, Carlos Manuel Benites, Patricia J. Garcia, Evelyn Hsieh

**Affiliations:** 1 Department of Medicine, Division of Rheumatology, Johns Hopkins University, Maryland, United States of America; 2 Department of Medicine, Section of Rheumatology, Allergy & Immunology, Yale School of Medicine, Connecticut, United States of America; 3 Yale School of Public Health, Connecticut, United States of America; 4 Epidemiology, STD, and HIV Unit, School of Public Health, Universidad Peruana Cayetano Heredia, Lima, Peru; 5 Epidemiology and Microbial Disease Department, Yale School of Public Health, Connecticut, United States of America; 6 Department of Internal Medicine, Waterbury Hospital, Connecticut, United States of America; 7 National HIV, STI and Hepatitis Program, Ministry of Health, Lima, Peru; Moi University College of Health Sciences, KENYA

## Abstract

As the population of persons living with HIV (PLWH) ages, the Peruvian National HIV, STI and Hepatitis Program (NHSTIHP) faces the question of how best to provide care for non-communicable diseases (NCDs) among PLWH. Using a qualitative approach, this study explores the barriers and facilitators of providing NCD care to PLWH in Peru through the perspective of NHSTIHP physicians and coordinators. HIV regional program coordinators and physicians affiliated with Peru’s NHSTIHP were recruited for individual in-depth interviews from the Program’s physician and coordinator registry and by peer referral. Interviews continued until thematic saturation was reached in both groups. The emerging themes were visualized using an adapted socioecological framework. All regional coordinators who volunteered (N = 25) were interviewed. Physicians were sampled to ensure variability in training background and region, and 14 physicians were interviewed to reach thematic saturation. Barriers and facilitators discussed by both groups were organized into major themes following a socioecological model: patient, provider, local clinic/hospital, regional, and national health system. Peru-specific themes identified included: concern about practicing outside of one’s specialty (“intrusismo”), patient trust, the role of HIV coordinators and insurance, and the importance of national clinical practice guidelines. This study highlights the complex interplay between patient, provider, local, regional, and national factors that influence delivery of care to PLWH in Peru. Addressing the barriers will require co-creation of resource-adaptable national HIV guidelines, with the participation of providers, program coordinators, and patients. These guidelines should encompass not only NCD management and educational resources for HIV providers and patients, but also processes and implementation pathways. This will require coordination between existing NCD and HIV health programs, and generation of epidemiologic data on NCD in PLWH to optimize resource allocation.

## Introduction

With tremendous strides in diagnostics and therapeutics for HIV since the 1990s, persons living with HIV (PLWH) can now expect a relatively normal life expectancy and we are seeing a demographic shift into older age worldwide [1,2]. Aging with HIV, however, presents its own unique challenge. PLWH have an increased risk of many non-communicable diseases (NCDs) due to a combination of traditional risk factors such as age or diet, and the dual impacts of chronic viral infection and antiretroviral therapy (ART) [3,4]. As the global HIV population ages, it is increasingly common that PLWH have multiple NCDs, and their healthcare services must also adapt to effectively manage this trend [5].

When examining the patient experience of living with NCD and HIV, patients have noted challenges with dysfunctional referral systems, poorer rapport with non-HIV specialists, and the burdensome cost of care [6]. As opposed to sending a patient on a specialist scavenger hunt for piecemeal management of their various disease processes, integrating NCD management within routine HIV care is a much more patient-centered approach [6]. Integration of NCD and HIV care services, however, is no trivial task, and may require modification of existing HIV care delivery systems, provider education, and resource re-allocation. Organizations worldwide endorse the incorporation of aging-related chronic disease management into HIV care [7], but there are few guidelines for how to best do so. Without clear existing strategies to address the need for longitudinal NCD care in their populations of PLWH, countries and health systems have tried various strategies: the strategy of integrating specific NCDs into existing HIV health infrastructure [8–12], the strategy of creating a hybrid NCD-HIV care center [13–17], and the strategy of bringing HIV/ART services into existing primary care infrastructure [18–20]. There is currently no official strategy employed in Peru to address NCD care for PLWH and most literature on various strategies comes from Africa or high-income countries (i.e., USA, UK) [8–20].

Between 2010 and 2020, the percent of new cases of HIV in persons over the age of 50 in Latin America increased by 30%, from 6.3% to 8.6% [21]. Within a large Caribbean Central and South American multicenter network (CCASnet), Peru had one of the fastest upward trends in the relative proportion of PLWH over the age of 50 over their 15-year study period [22]. In the Peruvian general population, seven out of ten deaths are attributed to NCDs [23]. A small but growing number of studies have begun to characterize the epidemiology of NCD in PLWH in Peru, however similar mortality/morbidity data are lacking. We previously surveyed HIV physicians across Peru and found that most are encountering and managing NCD to some degree among their patients with HIV [24]. However, they frequently reported lacking confidence in their knowledge of best practices for NCD care [24]. To better understand the context for these findings from the perspective of the Peruvian National HIV, STI and Hepatitis Program (NHSTIHP) coordinators and HIV providers, we conducted a qualitative study to explore the barriers and facilitators to providing NCD care to PLWH in Peru.

## Methods

### Ethics

The study was approved by the Ethics Review Committee of the Universidad Peruana Cayetano Heredia (#206475) on September 8^th^ 2021 and issued an exemption from Yale University’s IRB (#2000031131) on October 11^th^ 2021. Participants were contacted and recruited starting November 25^th^ 2021 through June 30^th^ 2022. Study participants provided verbal consent by telephone prior to participation in any study activities as approved by the IRB. Verbal consent was re-confirmed over Zoom and witnessed by two researchers. A written copy of the consent and study description was sent to participants. All participants who volunteered were interviewed via Zoom by a trained Peruvian research assistant and the lead investigator.

### Study setting

Access to health services in Peru depends on an individual’s insurance, financial resources, and geographic location within the country. The majority of PLWH in Peru are covered under Seguro Integral de Salud (SIS), a government insurance program funded by general taxes, which provides healthcare for those in poverty. There are three main levels of health care centers in Peru: the primary health centers, the small hospitals, and the large tertiary care hospitals. Half of the primary health centers are not staffed by a physician, and most do not have internet or adequate infrastructure [25]. Less than one quarter of Peruvians go to a primary health center first for a health need, the majority instead go directly to hospitals or purchase medications at pharmacies without a physician prescription [26].

The Ministry of Health’s NHSTIHP program provides care for approximately 77% of PLWH in Peru through a network of HIV providers and regional program coordinators [27]. These physicians are the primary point of medical care for PLWH in Peru, and like many lower-middle income countries, there is not a robust alternative primary care system or sufficient subspecialists to whom patients can be referred for NCD prevention and management. Regional program coordinators (one per region) are tasked with budget allocation and supply/medication requests, supervising region-specific HIV programming/community education, training staff on practice guidelines, and ensuring care delivery is meeting national metrics. There are regional coordinators for many other programs run by the ministry, such as for pregnant women/children or for non-communicable diseases, but coordination between programs is rare. Interviews discussed pre- and peri-pandemic health system conditions to contextualize the emerging themes and look towards a post-pandemic future.

### Study recruitment and participants

All HIV regional coordinators and HIV physicians working for the Ministry of Health’s NHSTIHP were potentially eligible for participation. Prior to the start of recruitment, an email from the NHSTIHP was sent to all HIV program coordinators notifying them about the study. Each regional HIV program coordinator was subsequently contacted by a trained Peruvian research assistant via WhatsApp and email who provided the coordinator with information about the study purpose and procedures, and a copy of the consent form. All 31 regional coordinators were invited to participate in a semi-structured in-depth interview. All 25 who agreed to participate were interviewed over Zoom.

HIV physicians for this study were recruited from participants enrolled in a national telephone survey study of 78 Peruvian HIV providers carried out by our team. Details regarding the parent study have been previously published [24]. In brief, HIV providers were identified via the NHSTIHP’s physician registry and through referrals from regional HIV program coordinators during the coordinator interviews. HIV providers working with the NHSTIHP are physicians trained as either infectious disease doctors or generalists, rarely with additional training in adult internal medicine. NHSTIHP physicians were eligible to participate if they had seen at least one patient with HIV within the past year, and if they currently worked at a SIS center. At the end of the telephone survey, all participants were asked if they would be willing to participate in a semi-structured in-depth interview over Zoom. Seventy of the 78 expressed willingness to participate in the in-depth interviews, and this group of physicians was then purposefully sampled to ensure adequate variability in training background and regional representation from the jungle, highlands, and coast.

### Data collection

All 25 regional coordinators who agreed to participate were interviewed over Zoom. In the physician group, the semi-structured interviews continued until thematic saturation was reached. All interviews were approximately one hour in length and had questions specific to the roles and experiences of each group (S1 Text). Physician and coordinator interviews discussed NCD management in general, however participants were prompted to talk in greater depth about common NCDs with which physicians were comfortable (i.e., diabetes) and uncomfortable (i.e., osteoporosis) as identified from the preceding published telephone survey study of this physician population, which covered seven NCDs [hyperlipidemia, hypertension, diabetes, osteoporosis, sarcopenia, non-AIDS defining cancers, neurocognitive impairment (NCI)] and three modifiable risk factors (obesity, tobacco, and alcohol use) [24] (S1 Text). The audio recordings from the Zoom interviews (recorded on Zoom) were transcribed using Sonix.ai. Transcripts were checked for accuracy by the Peruvian research assistant. The Spanish transcripts were translated into English following a standardized protocol by a team of bilingual translators. Codebooks were developed for the coordinator and physician interviews by consensus with the coding team. Each English transcript was coded by two independent coders (RS, DG, JH, DC), at least one of whom was Peruvian to ensure culturally appropriate codes. All codes were discussed, and discrepancies were resolved by consensus. Coded transcripts were uploaded into ATLAS.ti for thematic analysis.

### Thematic analysis

Because of the lack of existing data on this subject area in Peru, the qualitative portion of our study was conducted using an inductive approach and the data was analyzed with what Varpio et al. refer to as “theory-informing inductive data analysis” [28]. This is one method within the inductive approach to qualitative research, which begins with a desire to understand or explore a phenomenon and follows a grounded theory approach. This iterative process consists of initial, intermediate, and advanced rounds of coding [29]. Initial coding generates codes and begins to categorize them by looking for patterns and themes within the data [29]. Intermediate coding builds connections between categories, and advanced coding generates theory to explain the codes and categories [29].

The initial generated coding categories touched on factors that impact all levels of the health system from the patient to provider to the local health system infrastructure to the national health system. The emerging themes and subthemes were visualized using a socioecological framework. In the 1970s, Bronfenbrenner originally described the socioecological model as a conceptual theory that health and human development are influenced by many factors from individual to societal [30]. This model was further adapted by McLeroy in 1988 as a framework through which to understand health-related behavioral change, then later adapted to understand health care delivery barriers, and to help inform public health interventions [31–34]. Based on the ways in which this theory has been applied in the literature, the specific levels themselves are less important than the concept that individual behavior is the result of a complex system of interconnected influences [30–36]. Because the emerging themes from the initial coding rounds pertained to both individual, social, environmental, and political themes, it became apparent that the interaction between the individuals and their environment underpins the emerging themes and is the key to understanding the barriers and facilitators at work. For this reason, the socioecological framework was chosen to help visualize the generated theory.

## Results

Twenty-five regional coordinators and 14 physicians were interviewed to reach thematic saturation. The average age of coordinators was 46 + /- 9.1 years, the majority identified as female (72%), were trained as nurses or midwives (92%), and were from the coastal regions (52%). The average age of physicians was 46 + /- 9.5 years, and the majority identified as male (64%), were infectious disease trained doctors (86%) and were from the coastal regions (50%) (Table 1).

**Table 1:** Participant demographics.

	Coordinators N = 25	Physicians N = 14
**Age (mean) +/- SD [range]**	46 + /- 9.1 [31-65]	46 + /- 9.5 [32-59]
**Sex N(%)** **Male** **Female**	7 (28%) 18 (72%)	9 (64%) 5 (35%)
**Region N (%)** **Coast** **Highlands** **Jungle**	13 (52%) 8 (32%) 4 (16%)	7 (50%) 4 (29%) 3 (21%)
**Professional Training N(%)**	RN/Midwife 23 (92%) MD 2 (8%)	Infectious Disease 12 (86%) Generalist/Internist 2 (14%)
**Physician Practice Level N(%)**		Primary Health Center 3 (21%) Small Hospital 8 (57%) Tertiary Hospital 3 (21%)

The barriers for integrating NCD-HIV care that were identified through thematic analysis of the interviews were: HIV stigma, lack of provider NCD knowledge, training and comfort, limited appointment time, concern about practicing outside of one’s specialty (“intrusismo”), high cost of care and geographic access barriers, resource limitations, job turnover and politics, inflexibility of SIS and other hospital protocols, a lack of comprehensive NCD care guidelines for PLWH, a lack of NCD data or unified records systems, and fragmented public health systems with poor communication between NCD and HIV care efforts and departments.

The facilitators for integrating NCD-HIV care identified through thematic analysis of the interviews were: an awareness among providers of the growing issue of NCDs and a desire to improve the quality of care for PLWH, providers who are already undertaking NCD care for their patients with HIV, existing coordinator networks, existing NCD programming for the general population, existing HIV national guidelines, the trust PLWH have in their providers, and the patient preference for comprehensive care.

### Leveraging the socioecological model to understand the barriers and facilitators

**Fig 1:**
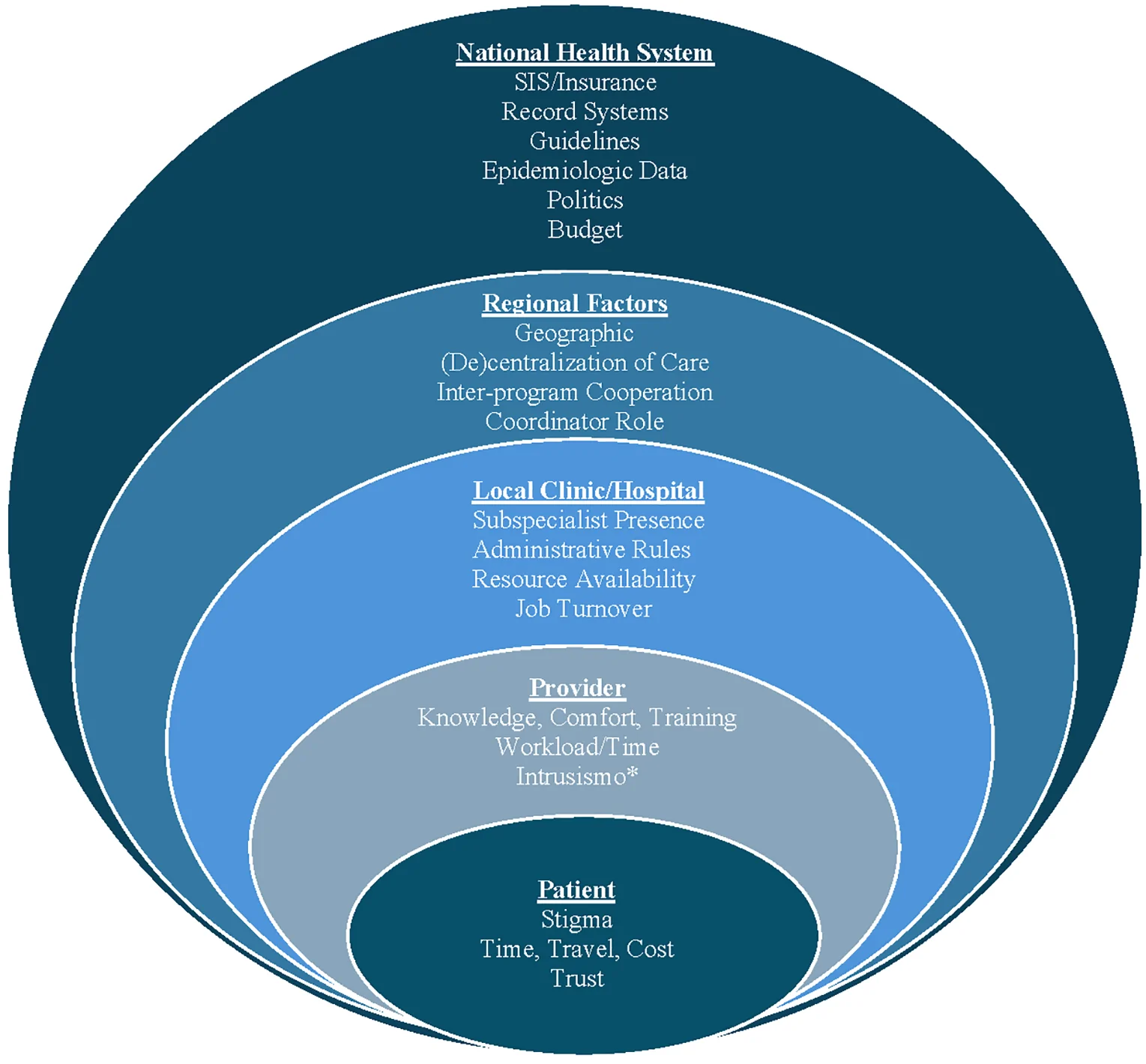
Socioecological Model of the Influences Impacting NCD care for PLWH. *Intrusismo translates to the concern about practicing outside of one’s specialty.

The barriers and facilitators discussed by both groups were organized into major themes following a socioecological model illustrating the interwoven influences at the patient, provider, local clinic/hospital, regional, and national health system levels (Fig 1, Table 2).

**Table 2:** Socioecological framework themes illustrated with representative quotes translated to English.

Level	Quote
**Patient/** **Society**	**Trust and Patient preference:** “it is precisely the patients with HIV who, once they trust their treating physician, will consult you about everything that happens to them. No matter how many times you tell them if you have a digestive problem, go to the gastro, if you have a headache, go to the neurologist, you are the first one they will talk to about anything that happens to them.” – Physician
	**Stigma**: “If a [leader] finds out that an indigenous community member is, of his community he’s [with HIV] they kick the person out, right? In the best scenario, they kick the person out. In the worst scenario, the person disappears. So we also can’t disclose the (status).” – Coordinator
	**Cost/Travel:** “From Campanilla to Juanjui it is a 4 or 6 hour boat trip.”– Coordinator
**Provider**	**Knowledge, Comfort, and Training**: “Like if I have a patient with diabetes and HIV, I don’t dare to give him insulin because I don’t know how to dose it, I don’t know if maybe I’m giving him too much or too little. […] There are medications that we cannot give and since we do not know how to give them.” – Physician “In patients with HIV we manage everything, hypertensive patients…[…] Oh, dyslipidemia too, right? They are so frequent that we handle them ourselves and sometimes when they are very complicated […] .we talk to the specialists” -Physician
	**Workload/Time:** “The time in the consultation is very short. Um, sometimes I wish I had more time to be able to talk, because doing every test takes time.” -Physician
	**Intrusismo:** “we also have to be careful about “intrusismo”, don’t we? [...] Branches that are not really within our competence. The “Colegio Médico” [Medical Association of Peru] is monitoring jealously to see who is doing “intrusismo”, doing other things for which they have not […] not been trained, right?” --Physician
**Health Center**	**Subspecialist Presence/Health Center Protocols:** “in Ayacucho, that (due to) the fact that there were no specialists, I ended up medicating (managing) more things that sometimes had nothing to do with my specialty.” – Physician
	**Resource Limitations**: “For example, in the hospital there is no place to, to do bone densitometry, right? So no, no, we don’t do screening.” --Physician
	**Job turnover:** “Each new professional) has his/her own learning curve. And again, sometimes unintentionally or […] without realizing it, they segregate, discriminate, stigmatize patients.” --Physician “Time goes by and [patients] lose trust because of the high turnover of the staff”-- Coordinator
**Regional**	**Lack of inter-departmental communication:** “There is another strategy [department] that manages noncommunicable diseases. But there is no coordination to see what the trend is in specific population as a population living with HIV. […] Almost everyone is on their own” – Coordinator
	**Geographic barriers:** “But we have to understand that the [Amazonian] communities are also quite far away. We are talking about five, six, seven hours from a (health) facility, let’s say (from) the referral center, right?” – Physician
	**Decentralization efforts:** “Before there were only hospitals, nothing else, that gave HAART. […] Now more than 61 health centers throughout Metropolitan Lima and Callao provide HAART, right? So the decentralization of HAART has been very good.” –Physician
	**Coordinator Role:** “When a problem arises, mmm serious (problem), they come here, talk to me and we try to correct the problem with the hospital or with the health establishment that is providing them antiretroviral drugs so that they don’t feel marginalized” – Coordinator
**National**	**Insurance/SIS limitations:** “SIS does not recognize the patient, right? It does not recognize him. Since he is from Callao (his SIS) is not recognized in Lima.” --Coordinator
	**Need for guidelines:** “I do think that a guideline could be established, a guideline could be.... Updated obviously on non-communicable diseases in HIV patients. […] So, a guide would help us a lot so that the infectious disease physician can also enter this field and contribute to the timely detection of cases in order to prevent these diseases from continuing, continuing to advance.” -Physician
	**Need for records systems:** “it will be great if the patient has an electronic medical record linked to their DNI. We are wasting time and supplies because of this issue.” -Coordinator
	**Lack of epidemiologic data:** “we see them [NCDs] very little maybe because we don’t look for them” --Physician
	**Budget constraints:** “Every year the same amount of money comes and instead of increasing, it decreases. So how do you want to get more things done? No it cannot be done.” -- Coordinator
	**Politics:** “[Regional] Directors are also changing, sometimes we have 4–5 per year, so it can be also tricky to teach them every time. Moreover, sometimes these positions are “trust position” [positions given to supporters], so there are some politics involved.” --Coordinator

### Influences at the patient/society level

Within the patient and society level of influence, the major themes discussed by physicians and coordinators were trust, the burdens of care access (cost, time spent, and travel distances), and HIV stigma. Physicians noted that they had a unique opportunity of being in a position of trust with their patients and as a result, they were the first people their patients talked to about any health issues, whether they were related to HIV or not:

“*The HIV patient gets older. The HIV patient is going to have his genetics, his heredity. They have eating habits or when they feel well they will eat everything. So all those factors are going to make his predispositions awaken. And let’s remember that those of us who work with PLWHA have that great advantage that the first person they are going to tell is us. We should not miss that opportunity.” --Physician*

As a result of the trust built in these patient-physician relationships, our interviewees reported that their patients frequently wanted their HIV provider to be their “medico de cabecera,” or primary doctor, who handled all of their HIV and NCD diagnoses. Our interviewees explained that this desire for comprehensive care from one trusted provider also stemmed from a desire to avoid wasting time traveling to multiple subspecialists and the costs associated with that travel and time off work, which was sometimes prohibitive when added to the cost of their medications and healthcare.

A balancing force to comprehensive care was the stigma patients encountered from society and from health care professionals. Interviewees noted patients were hesitant to disclose their status to new providers, to the extent that they did not want their HIV provider to place a referral for specialized care because it would mean other providers would know about their HIV diagnosis. Sometimes patients preferred to travel to a further clinic or region for HIV care to avoid people in their community knowing their HIV status. This both drove patients to want their HIV physician to provide all their care, but also sometimes limited what referrals they felt comfortable with their provider placing:

*“Many times, our PLWH don’t want that any other person to know their diagnosis, sometimes they also don’t want to have a referral when they have diabetes or hypertension. They get an appointment on their own and go as general population.” -- Coordinator*

### Influences at the provider level

While empathetic to their patient’s desire for them to provide comprehensive NCD care in addition to HIV care, physicians noted barriers of knowledge and training, comfort and interest, time/workload, and a concern that they would be overstepping the bounds of their scope of practice.

While some HIV physicians often felt comfortable managing common metabolic diseases such as diabetes, hypertension, and hyperlipidemia for their HIV patients, others felt that they did not have the knowledge or training to go beyond HIV care. Several physicians noted that even if they wanted to, there was simply not enough time in a patient encounter to address all the necessary NCDs in addition to HIV. Increasing workload may risk overburdening staff to the point of providers leaving their jobs. In addition to the loss of providers leading to reduced care access, coordinators noted that new HIV providers had “his/her own learning curve” with respect to providing HIV care and new staff may, even inadvertently, be a source of HIV stigma and discrimination.

Despite the workload pressures, many noted that they needed to be involved in NCD care to at least some degree for their patients with HIV because they had unique insight into the interaction of HIV, antiretroviral therapy, and their patient’s lifestyle.

*“There’s a difference that when they go to the [primary] health center  ... [...] They do not say their HIV status. [...] Suddenly, the general practitioner treats the non-communicable disease in isolation. When many times, let’s be honest, metabolic disorders can be due to antiretroviral treatment. [...] So, if you don’t make that connection, the treatment of the problem is not going to be ideal.” --Physician*

That said, some physicians worried that being involved in NCD care was stepping into the territory other specialties (“intrusismo”). They reported that their ordering and prescribing practices were monitored at the local and national level. When asked what the result of this oversight was if they overstepped, physicians spoke of getting unwelcome scrutiny by administrators, which served as a deterrent from going beyond their scope of practice.

### Influences at the health center level

Administrative protocols and the resources available at the health center had a large impact on physician practice. At primary health centers and the secondary level hospitals, access to screening tests and medications were limited. Depending on the region, even the largest hospitals lacked certain screening tests like mammography for breast cancer or bone densitometry for osteoporosis. At well-resourced sites with more specialists and more resources, there were sometimes more administrative protocols about which physicians could order which tests.

Some physicians reported that they could provide NCD care for diseases they viewed as less common in their population, such as osteoporosis, depending on whether they had anyone to refer their patients to. When there were few specialists to assist with NCD care, HIV providers might attempt to manage a variety of NCDs for their patients:

*“If there is no rheumatologist, the only one who is a doctor would be the only one who could treat it properly. […] So, in those cases, yes, I would dare to manage, right? I would try to learn, to talk with another colleague (about) what would be good for this type of patient.” –Physician*

Coordinators and physicians also pointed out that job turnover of physicians and coordinators, especially in the more remote clinics and hospitals resulted in newcomers who needed time and training to become comfortable in the system. Job turnover of physicians and coordinators eroded patient trust and brought stigma in the form of new providers who were not comfortable yet with caring for PLWH.

### Influences at the regional level

The regional coordinators were motivated to improve care for PLWH. They highlighted influences such as the lack of inter-departmental communication, trends towards decentralization, and geography. In many regions, coordinators noted that there was a non-communicable disease “strategy,” which had its own regional and local coordinators, budget, and priorities. Despite often sitting in the same regional office building, there was little to no communication or shared goals between the HIV strategy and the NCD strategy coordinators.

As a geographically diverse country with communities living in the highland peaks or the Amazon jungle, accessing specialized care and HIV care often meant traveling miles on foot or by boat to the major cities in each province. Across all the regions, coordinators reported trends towards decentralization of antiretroviral therapy into primary level health centers to bring the medication prescribing closer to the patients and improve care access. While this has been successful in many cases, coordinators have faced push-back from the staff in certain health centers who are not comfortable with HIV care and may already have a high workload.

*“We have already told them that there are patients [with HIV] who are chronic, who are already stable, who have a base medication and who are doing very well and then they could be managed at the primary care. Then the refusal begins, because they consider that they are going to assume a very great responsibility. “What’s going to happen if they get complicated?” – Coordinator*

Despite efforts to improve NCD care access at the regional level, however, health resources are generally concentrated in the capital city of Lima. As one physician notes:

*“Whether you have HIV, whether you don’t have HIV, there are tests that are limited in certain regions.” – Physician*

### Influences at the national level

Just as budgets and health campaigns between different health departments remained separate within regions, on a national level, there was no program for comprehensive age-related care which coordinated with the NHSTIHP. Although many interviewees reported that they were aware of the growing burden of NCD in patients aging with HIV, they emphasized the lack of epidemiologic data to back up their observations. They pointed out the need to track and record NCD, but also noted that any data they already had may be underreported because screening is not always performed, and data is easily lost in paper medical records.

Although SIS, the public health insurance, is a national system, it is geographically tied to a patient’s officially documented region of residence. For example, if a patient travels to a different region, they might not be recognized as being enrolled in the public health insurance when trying to access care. These geographic limitations of SIS create barriers for patients, especially those who travel to work in other regions or wish to go further away from home for their care due to HIV stigma.

Additional national level influences that were mentioned as barriers to care included national budgetary constraints and resource limitations, especially in the setting of the pandemic and the shift in health priorities that was necessary at the time. Interviewees also noted that politics on a national level could pose challenges to health service delivery. For example, political appointments to health leadership roles could be challenging if the appointee did not have the appropriate background or experience.

Both physicians and coordinators discussed the role of the “norma nechnica,” the existing national guideline for HIV care. The “norma technica” guides the coordinators and physicians on how to diagnose and treat HIV. It also includes some NCD care testing and screening, such as checking glucose and lipid levels, performing pap smears and mammography, but does not go into the details of follow up management for any NCDs beyond ordering the test. Physicians voiced a desire for guidelines for NCD care for PLWH.

*“A guide would help us a lot so that the infectious disease physician can also enter this field and contribute to the timely detection of cases in order to prevent these diseases from continuing, continuing to advance.” -Physician 105*

That said, both coordinators and physicians emphasized that a guideline alone without efforts to train and motivate providers would not be successful:

*“There’s so much paper, so much standard (guidelines), so many things that we should be doing in the strategy. I think in the end it all comes down to how interested everyone is in helping PLWH.” – Physician 126*

## Discussion

As the global population with HIV ages, NCD has become an inevitable part of living with HIV. Health care systems worldwide have been forced to wrestle with how to best provide care for persons living with the dual burdens of HIV and NCD [37,38], but most of the existing literature is from outside Latin America. This is the first study to assess the barriers and facilitators to providing NCD care for PLWH in Peru. We identified key perspectives that have not been previously described in the literature, such as physician hesitancy to overstep their scope of practice (“intrusismo”), the rigidity of the national insurance program in dictating care, and provider time and workload constraints. We highlight the significance of patient trust in their providers and the unique role HIV physicians feel they have as the primary point of medical care for their patients. We illustrate the important role that regional coordinators and national HIV guidelines serve in setting and disseminating standards of HIV care in Peru.

We also found that provider knowledge and training, stigma, resource access, and lack of epidemiology data were important influences and often key barriers to providing NCD care for PLWH in Peru, as have been mentioned in similar qualitative work from Sub-Saharan Africa [15,19,36]. Like most countries, HIV-related stigma persists both within the medical system and patient communities in Peru. PLWH may fear disclosure to their communities and sometimes seek care further away from home than they might normally to avoid disclosure of their HIV status.

As our prior study highlights, incorporating basic NCD care into the existing care provided by HIV physicians is already happening in many cases, but there is a lack of uniform NCD practice patterns, comfort and knowledge among HIV providers [24]. From our findings, we theorize that expanding existing national HIV guidelines to cover basic NCD management with flexible strategies to address different resource settings, may alleviate some of the resource barriers, scope of practice concerns, and the administrative barriers imposed by insurance and clinical sites that follow national guidelines to dictate ordering/prescribing practices. These guidelines would ideally include both initial basic management recommendations for common NCDs and outline practical workflow and implementation strategies.

In parallel, co-creation of educational resources with HIV physicians, coordinators, and patients may increase physician comfort with basic NCD care, decrease stigma, and leverage the trust within the established HIV physician-patient relationships. Facilitation of joint programming across NCD and HIV health departments may provide cohesive, multi-disciplinary, age-related care, and utilize existing health infrastructure and partnerships. Obtaining epidemiologic data on NCD in PLWH is necessary to determine areas of highest prevalence and morbidity/mortality and to provide cost and resource-effective guidance for patients and providers.

The majority of HIV physicians, regardless of their professional training, work for the Ministry of Health via the NHSTIHP so interventions from the NHSTIHP will have a wide-ranging impact in Peru. As we have learned from our study however, any efforts towards NCD integration must be done thoughtfully, guided by epidemiology to target the most impactful interventions. Our socioecological framework emphasizes that stand-alone interventions will not be as effective as a systematic approach. The identified barriers and facilitators cannot be thought of as individual components of an issue, but rather as interrelated influences within a complex system. For example, our participants report that national policy to expand HIV guidelines to include NCD basics is important in enabling comprehensive NCD care for PLWH. From the socioecological framework, we see that a single intervention such as expanded national HIV guidelines alone will not be successful in isolation without simultaneously addressing the time constraints of a routine patient visit, the cost of care, insurance limitations, testing/screening and specialist resource access, provider knowledge, training, and comfort enacting recommendations, and how stigma impacts where and how patients access care. Overburdened staff without access to knowledge, training or resources to provide the care they are asked to provide may leave their jobs. In addition to decreasing health care access, high job turnover may in result in increased discrimination as HIV providers who are unaccustomed to caring for PLWH may also, often without realizing, contribute to patients feeling stigmatized in the medical system.

Our interviews highlight the ongoing efforts across the country to decentralize HIV care from the secondary and tertiary hospital-based care centers into the more widely accessible and lower resourced primary level clinics to increase patient access to antiretrovirals and alleviate geographic and mobility barriers. Although the COVID-19 pandemic accelerated this process, much of HIV care is still performed by infectious disease trained physicians in secondary and tertiary hospitals. Incorporating HIV care into routine primary care will become increasingly important as decentralization efforts continue, but will require strengthening of the current primary care infrastructure, increasing access to HIV screening tests, and HIV-specific training for generalists and internists. Constructing separate NCD-HIV hybrid clinics, like Shayo et al. and Akugizibwe et al. have tried in Africa would not make sense in Peru where the prevalence of HIV is low in the general population [14,15]. If resources allow, a regionally-based rotating mobile specialist model like Wroe et al. proposed in Malawi may be a way to “decentralize” subspecialist care into the primary health level until primary level providers are upskilled [12,13], but geographic limitations (i.e., mountainous terrain, lack of roads) may limit the reach of such a model in Peru.

This study had some important limitations. Because our data is grounded in the Peruvian health system and culture, direct generalizability to other contexts is limited. Physicians and coordinators who agreed to participate in these interviews may have felt more passionately about the importance of non-communicable disease care. As such, this group may be more likely to favor integration of NCD and HIV care and feel more urgency about the necessity of engaging with NCD to improve their patients’ quality of life. Although our interviewees represent two important perspectives on how to provide NCD care to PLWH, it will be essential to obtain opinions from other key stakeholders such as the patients themselves, clinical nursing staff, and policy makers. In addition, it is important to acknowledge that any interpretation through translation carries the risk of misinterpretation both linguistically and culturally. In any country, the medical system itself has its own culture and language. To minimize misinterpretations and misattributed meaning, we involved Peruvian translators and coders who were familiar with the Peruvian medical system at every step of the data collection and analysis. Further research on implementation and feasibility must be conducted prior to introducing health system level changes.

## Conclusion

The barriers and facilitators we have identified have greater meaning within the context of the health system and the various levels of influence at play. Using a socioecological model, our study highlights the complex interplay between patient, provider, local, regional, and national factors that influence NCD care delivery to PLWH in Peru. Strengthening the capacity of NHSTIHP HIV physicians and coordinators to provide basic NCD care by coordinating with existing regional NCD programs could leverage the existing health system resources in Peru. Addressing the existing barriers will require support from HIV physicians, patients, coordinators, and the national health system. Creating resource-setting adaptable national HIV guidelines, which include basic NCD management and educational resources for HIV providers and patients, and obtaining epidemiologic data on NCD in PLWH to optimize resource allocation will be crucial pieces in improving NCD care for PLWH. Although by nature our study is deeply rooted in Peru, we illustrate the importance of conceptualizing NCD care for PLWH as a systems-issue, best addressed on multiple levels, from patient to national, which is applicable to healthcare efforts at mitigating the growing burden of NCD among PLWH worldwide.

## Supporting information

S1 Text Interview guides. (DOCX)
