# Supplementary material for: Exploring barriers and facilitators for non-communicable disease care for persons living with HIV in Peru through a socioecological framework: HIV physician and coordinator perspectives
Source: PLOS Glob Public Health. 2026 Sep 25;6(9):e0006608. doi: 10.1371/journal.pgph.0006608 (PMC13614537; doi:10.1371/journal.pgph.0006608)
Supplement: Supplementary file 1 [file pgph.0006608.s001.docx]

**S1 Text: Interview Guides**

**Coordinator Interview Guide:**

I. **Open-ended questions/ Work as a coordinator**
1. Age:
2. Gender:
3. Profession :
4. Years in office, at MINSA:
5. Tell us about your role as coordinator:
   - What are your responsibilities?
   - What difficulties do you have in your role as coordinator? What makes it easier?
   - ***If appropriate: It's so nice to see how passionate you are about your work, what sustains your engagement in this job?***
6. How many doctors work in your Establishment or RED or DIRESA?
   - In general, what do you think are the main needs and obstacles faced by the doctors of your Establishment or RED or DIRESA for the management of PLHIV?
   - **If appropriate: can you tell me more about the relationship between the ministry and the NGOs?**
II. **About NCDs:**
7. Have you heard in your Establishment or NETWORK or DIRESA problems of non-communicable diseases in patients with HIV, e.g. hypertension, diabetes, osteoporosis?

Not 🡪 why?

If 🡪 and who manages their chronic problems? You know?

8. Do you think that the doctors of the Establishment or RED or DIRESA who treat patients with HIV should also be the ones who manage these non-communicable diseases? Why or why not?
9. Is there a program for the management and prevention of non-communicable diseases in general in your establishment or RED or DIRESA?
10. If your work included additional registration of noncommunicable diseases in HIV patients, what additional resources would you need to do this successfully?
    - Changes to the system or program you use for registration
    - Changes in the health personnel involved
    - Material Resources You Would Need
11. How did COVID-19 affect your work as a coordinator? In addition:
    - Have there been any changes in the number of patients seen by the doctors you coordinate?
    - Have there been any changes in the health care staff who manage patients with HIV?
    - Has health insurance been affected in any way for patients? Have there been any changes in the funding of your labor department?

**If at 45min at this point, consider gathering data (patient info and doctor's contact) via WhatsApp:**

III. **About Patients and Doctors**
12. What data or records do you work with? How do you keep your records: Excel, other? Who provides you with the information? How often? How many HIV patients do you have registered in your Establishment or RED or DIRESA?
    - How many in targa?

Do you have the age data? How many are over 40 years old?

- *Are HIV patients who had or have COVID registered? Why?*
  i. *Yes: How many of your HIV patients have had COVID?*
- *Do you register those who have been vaccinated? Why?*
  i. *Yes: How many have been vaccinated?*

13. With everything we have discussed in this interview, do you have any additional comments regarding the management of HIV patients or your work as a coordinator?

**Physician Interview Guide:**

I. **Context:**

| *Infectious Disease Specialists* | How does it feel to work with HIV patients? What's the most challenging? |
| --- | --- |
| *General Practitioners Who Became Specialists* | How has your experience as a general practitioner affected the way you manage your HIV patients?  *(We are interested in how you went from general practitioner to specialist, was it through any* particular application process*?)*  How does it feel to work with HIV patients? What's the most challenging? |
| *General practitioners or internists* | How did you get started working with the strategy?  How has your experience as a general practitioner affected the way you manage your HIV patients?  How does it feel to work with HIV patients? What's the most challenging? |

II. **The system:**
1. Overall, what do you think is the ideal way to provide NCD care to your HIV patients?

*Next: 🡪* For you in your establishment? Through other specialists?

What preferences have your patients expressed regarding where/who consults for their NCDs?

III. **NCD Specifics - Musculoskeletal diseases:**

From our survey, we saw that many doctors are less comfortable managing musculoskeletal diseases such as osteoporosis and sarcopenia. We would like to use these as an example for the following questions.

1. How do you identify patients in your facility at risk of osteoporosis or sarcopenia for screening?

*If so🡪* , will you know how that consultation went? Will you know if patients encounter stigma when consulting other specialists?

2. Can you prescribe bisphosphonates through the SIS? A densitometry? (other screening tools?)

*(If they are infectious disease specialists🡪)* Have you noticed limitations with the SIS when infectious disease specialists order tests to detect non-communicable diseases in general? For example, diabetes?

3. How have you been training (learning?) on these topics, osteoporosis or sarcopenia? For HIV patients and in general? Does this training help with your current practice? *(e.g....)*
4. What would help you manage osteoporosis or other noncommunicable diseases in your HIV patients? For example? Would a guide and/or a mobile app help?
5. Do you have any other additional thoughts or comments on noncommunicable disease management in PLWH that we haven't discussed so far?
